# Rhoptry Proteins ROP5 and ROP18 Are Major Murine Virulence Factors in Genetically Divergent South American Strains of *Toxoplasma gondii*

**DOI:** 10.1371/journal.pgen.1005434

**Published:** 2015-08-20

**Authors:** Michael S. Behnke, Asis Khan, Elvin J. Lauron, John R. Jimah, Qiuling Wang, Niraj H. Tolia, L. David Sibley

**Affiliations:** Department of Molecular Microbiology, Washington University School of Medicine, St. Louis, Missouri, United States of America; Fred Hutchinson Cancer Research Center, UNITED STATES

## Abstract

*Toxoplasma gondii* has evolved a number of strategies to evade immune responses in its many hosts. Previous genetic mapping of crosses between clonal type 1, 2, and 3 strains of *T*. *gondii*, which are prevalent in Europe and North America, identified two rhoptry proteins, ROP5 and ROP18, that function together to block innate immune mechanisms activated by interferon gamma (IFNg) in murine hosts. However, the contribution of these and other virulence factors in more genetically divergent South American strains is unknown. Here we utilized a cross between the intermediately virulent North American type 2 ME49 strain and the highly virulent South American type 10 VAND strain to map the genetic basis for differences in virulence in the mouse. Quantitative trait locus (QTL) analysis of this new cross identified one peak that spanned the *ROP5* locus on chromosome XII. CRISPR-Cas9 mediated deletion of all copies of *ROP5* in the VAND strain rendered it avirulent and complementation confirmed that *ROP5* is the major virulence factor accounting for differences between type 2 and type 10 strains. To extend these observations to other virulent South American strains representing distinct genetic populations, we knocked out *ROP5* in type 8 TgCtBr5 and type 4 TgCtBr18 strains, resulting in complete loss of virulence in both backgrounds. Consistent with this, polymorphisms that show strong signatures of positive selection in ROP5 were shown to correspond to regions known to interface with host immunity factors. Because ROP5 and ROP18 function together to resist innate immune mechanisms, and a significant interaction between them was identified in a two-locus scan, we also assessed the role of *ROP18* in the virulence of South American strains. Deletion of *ROP18* in South American type 4, 8, and 10 strains resulted in complete attenuation in contrast to a partial loss of virulence seen for ROP18 knockouts in previously described type 1 parasites. These data show that ROP5 and ROP18 are conserved virulence factors in genetically diverse strains from North and South America, suggesting they evolved to resist innate immune defenses in ancestral *T*. *gondii* strains, and they have subsequently diversified under positive selection.

## Author Summary

Parasites and the hosts they infect are in constant struggle with each other for survival. On the one hand, the host needs to control parasite growth, while the parasite needs to evade the host response long enough to allow for efficient transmission. The parasite *Toxoplasma gondii* has evolved virulence factors ROP5 and ROP18 to evade innate immune mechanisms of its natural intermediate host, small rodents. These genes were initially identified in clonal parasite types isolated from Europe and North America, but the factors that contribute to virulence in genetically divergent South American strains have not been tested. Here we used forward and reverse genetic analyses to show that ROP5 and ROP18 are also major virulence factors in genetically distinct virulent South American strains. Given that ROP5 and ROP18 function as virulence factors in strains from North America, Europe, and South America they likely acquired their functions before *Toxoplasma gondii* radiated into its present global population structure.

## Introduction

Intracellular parasites have to contend with immune responses mounted by the hosts they infect if they are to survive long enough to effectively transmit. The parasite *Toxoplasma gondii* is one of the more successful parasites in terms of transmission as it can infect all mammals and many birds [1], both of which serve as intermediate hosts where the parasite propagates asexually. The parasite undergoes sexual recombination only within the intestinal tract of members of the Felidae family when these predators ingest chronically infected intermediate hosts [2]. Transmission by this predator-prey life cycle has shaped the co-evolution of parasite virulence factors and host responses.

*T*. *gondii* readily infects humans but mainly causes disease in situations where the immune system has become compromised, although associations between parasite genotype and disease severity may also occur in healthy individuals [3]. The association of virulence with parasite genotypes has been more fully investigated in laboratory mice, where this trait can be more easily assessed and where the parasite has likely had more opportunities to evolve within a host that is common prey of cats. Initially it was shown that parasite isolates from Europe and North America differed in their ability to cause disease in laboratory mice. Type 1 strains lead to lethal infection in all conventional strains of laboratory mice including outbred animals (LD_100_ = 1) during the first two weeks of infection (i.e. the acute phase), and any survivors observed at low inoculum are invariably not infected (i.e. they remain serologically negative) [4,5,6]. In contrast, type 2 strains show intermediate virulence (LD_50_ varies with mouse strain, although these strain generally do not cause mortality in outbred lines), and type 3 strains are avirulent (they are not lethal in inbred mice at any dose), respectively [4,5,6]. These phenotypic differences prompted the generation of genetic crosses between these three strain types, resulting in the identification of the rhoptry kinase ROP18 as a virulence factor in genetic crosses between type 1 and type 3 or between type 2 and type 3 strains [7,8], and the rhoptry pseudokinase ROP5 as a virulence factor in genetic crosses between type 2 and 3 or between type 1 and 2 strains [9,10]. Together these proteins are involved in resisting innate immune mechanisms that are induced by IFNg stimulation [11]. The parasite secretes ROP5 and ROP18 into the host cell during invasion where they co-localize to the surface of the parasitophorous vacuole [12]. ROP18 is tethered to the vacuole membrane by an N-terminal low complexity region [13] and this association is required for its virulence enhancing properties [14].

At the vacuole surface, ROP18 phosphorylates host immune-related GTPase (IRGs) and prevents them from accumulating on the membrane and destroying the parasite vacuole [12,15]. The association of ROP18 with ROP5 increases the phosphorylation activity of ROP18 [16], and may also make substrates available to the kinase by binding to members of the IRG family [17,18,19]. Biochemical studies subsequently showed that ROP5 also associates with ROP17, and that this kinase works synergistically with ROP18 to phosphorylate and block IRGs [20]. Deletion of either ROP17 or ROP18 alone leads to a modest attenuation in the type I RH strain, evident as a delay in time until death; however, mice still succumb to low challenge doses (i.e. 100 parasites). In contrast, deletion of both ROP17 and ROP18 leads a marked attenuation of virulence where mice survive high challenge does (≥ 10^5^ parasites) [20], similar to the deletion of ROP5 (≥10^6^ parasites) [9,10].

South American stains are much more genetically diverse than those in the North and our current estimate of the population structure consists of 6 major clades that contain a total of 16 distinct haplogroups [21,22]. Notably, most common haplotypes from South America are also virulent in mice [23], similar to the type I lineage of North America where this phenotype is otherwise rare. Importantly, South American strains also exhibit the trait that a single infectious organism is lethal in all conventional laboratory mice (i.e. LD_100_ = 1) and they do not give rise to serologically positive surviving animals [23]. Previous studies have shown that ROP5 alleles in North and South American strains are correlated with resistance to IRG coating of the parasitophorous vacuole [18] and that expression of ROP18 from the South American strain RUB (type 5) confers virulence to an avirulent type 3 parasite [24]. However, the precise contributions of ROP5, ROP18, or other genetic factors, to acute virulence in these strains are unknown. For that reason, we mapped the genetic basis for differences in acute virulence between the intermediate virulence type 2 ME49 strain and the highly virulent type 10 VAND strain using a recently conducted genetic cross [25]. The type 2 ME49 parental strain was isolated from a sheep in the USA [26], whereas the type 10 VAND parental strain was originally isolated from an immunocompetent human adult in French Guiana [27]. Using quantitative trait locus (QTL) mapping and CRISPR-Cas9 generated knockout (KO) lines we were able to identify ROP5 as the single major virulence factor underlying the virulence differences in laboratory mice between type 2 and type 10 parasites. Additionally assessment of the contribution of ROP18 to virulence in several South American strains revealed a stronger phenotype than has been previously described for North American type 1 strains. These findings demonstrate that the genetic basis of mouse virulence is conserved across diverse lineages of *T*. *gondii*.

## Results

### Virulence maps to the *ROP5* locus in the ME49-FUDR^r^ X VAND-SNF^r^ cross

**Fig 1:**
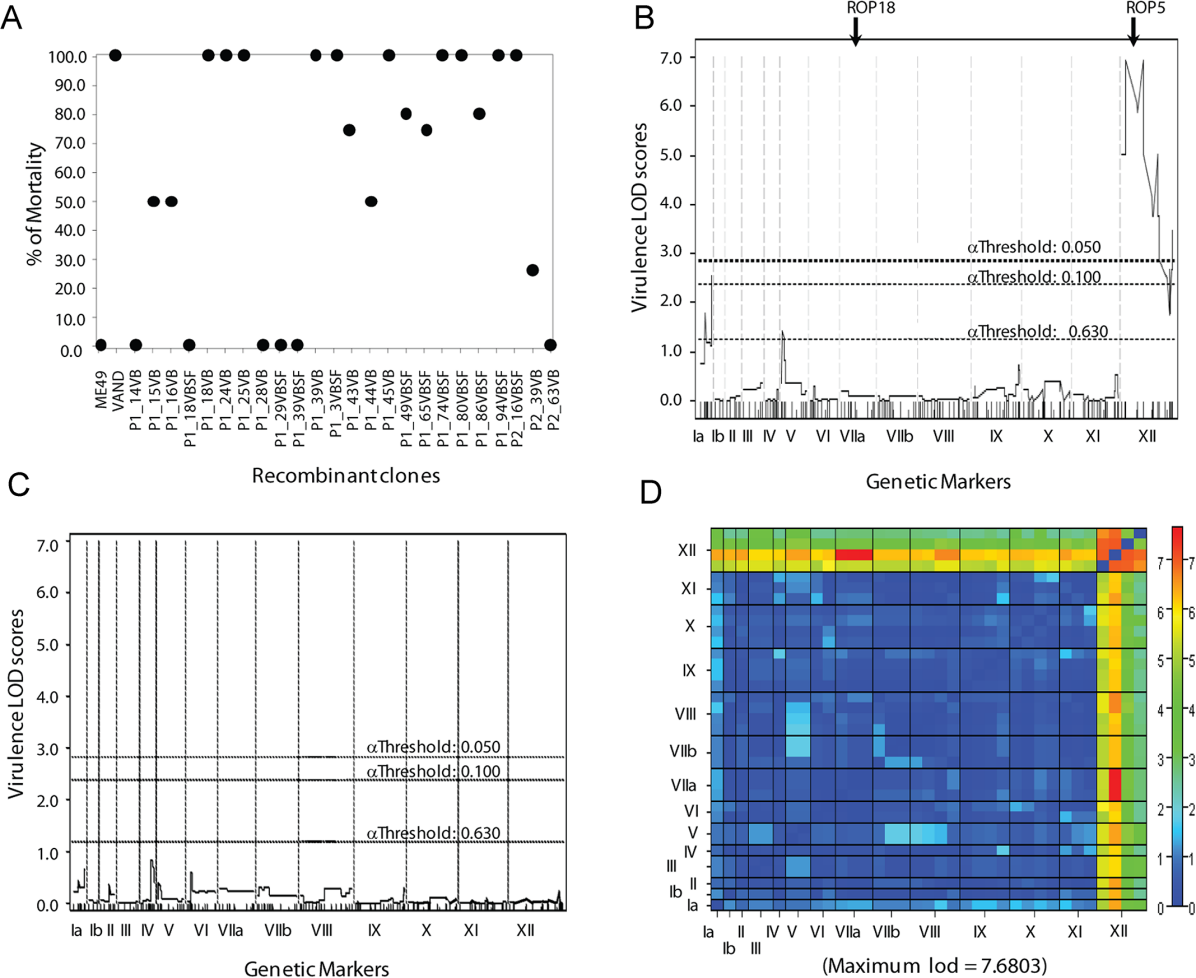
Genetic mapping of the virulence phenotype identified the ROP5 locus as the single major QTL. A) The parents and progeny of the genetic cross between type 2 ME49-FUDR^r^ and type 10 VAND-SNF^r^ strains were assessed for virulence in 5 outbred CD-1 mice per strain. (B) A QTL scan of the virulence phenotype generates one significant peak on the left end of chromosome XII with a LOD score of 6.95 and effect size 71%. Genome-wide LOD significance thresholds were obtained from 10,000 permutation tests, α .05 ≥ LOD 2.7. (C) A secondary scan of the virulence phenotype with the locus on chromosome XII run as an additive covariate. (D) A two-dimensional genome scan with a full model testing for interacting QTLs (top half) and an additive model testing for multiple QTLs without interaction (bottom half). Genome-wide LOD significance thresholds were obtained from 1,000 permutation tests.

We utilized a previously described genetic cross between type 2 ME49-FUDR^r^ and type 10 VAND-SNF^r^ strains [25,28]. Whole-genome sequencing of 24 progeny and mapping based on genome-wide SNP analysis was previously used to identify the molecular basis of sinefungin resistance [29]. Here we used the existing genetic map from this cross to analyze virulence of 24 recombinant progeny in CD-1 outbred mice. The parental ME49-FUDR^r^ strain generally does not cause lethal infection in CD-1 mice and 7 of the progeny inherited this trait, where most or all of the mice infected with these progeny survived the 30 day course of the experiment (Fig 1A). There were 14 progeny that acquired the virulent trait from the VAND-SNF^r^ parent, exhibiting ≥ 75% mortality in CD-1 mice (Fig 1A). Only three progeny showed an intermediate phenotype, with 50% of the mice succumbing to infection when infected with these progeny (Fig 1A).

A genome-wide primary QTL scan of virulence (% mortality) run as a continuous phenotype generated one significant peak between the physical locations of 0.48 MB and 2.94 MB on chromosome XII with a log_10_ of odds (LOD) score of 6.95 (Fig 1B). A second QTL was detected in the primary scan with a LOD score of 2.54 on chromosome Ia; however, this peak did not reach significance (Fig 1B). The single peak on chromosome XII accounted for 71% of the effect-size, or variance in the virulence phenotype. A secondary scan with the primary QTL on XII run as an additive covariate failed to produce additional significant peaks (Fig 1C). Although no secondary peaks were identified, analysis of the virulence phenotype using a two-locus model identified a significant interaction between the primary peak on XII and chromosome VIIa, with a LOD score of 7.68. This finding is of interest as the known virulence factors ROP5 and ROP18 are located on chromosomes XII and VIIa, respectively.

### Deletion of ROP5 in the VAND strain reduced virulence in mice

**Fig 2:**
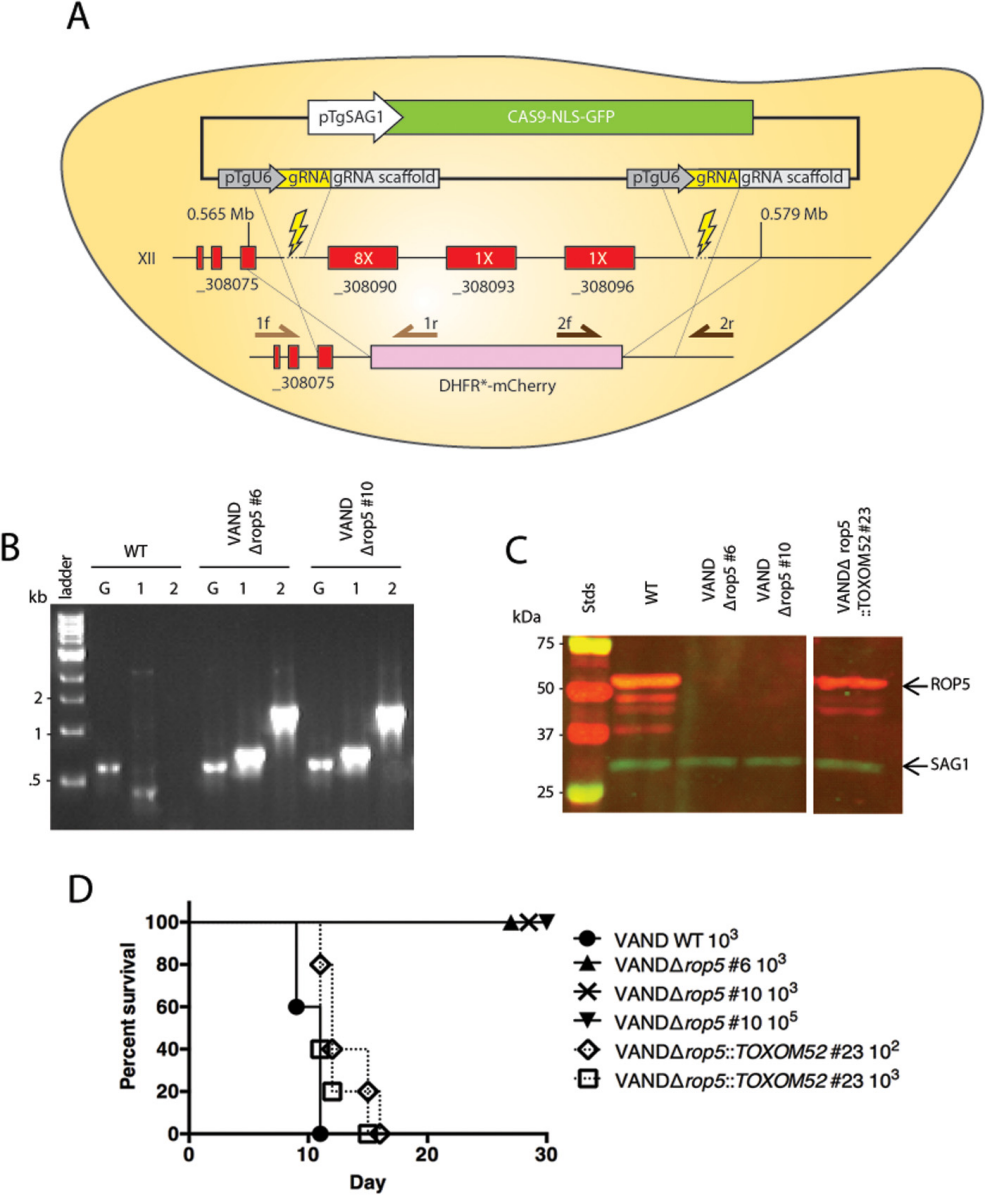
ROP5 accounts for virulence phenotype in the type 10 VAND strain. (A) Schematic diagram for knocking out the ROP5 locus on chromosome XII. Representation of the CRISPR-Cas9 plasmid (green box) and double CRISPR sgRNAs (yellow boxes), the sgRNA cut sites (lightning bolts), genes at the locus (red boxes) with copy number estimates of the ROP5 genes (white text in red box), the integration of the DHFR*-mCherry cassette (pink), and primers used in diagnostic PCR (arrows). (B) Diagnostic PCR showing integration of the DHFR*-mCherry cassette in two VANDΔ*rop5* clones, G: GRA1 promoter positive control—primer 1: 5’ integration—primer 2: 3’ integration—primer Set5-2. (C) Western blot of the VANDΔ*rop5* mutant and a complemented clone expressing the ToxoM52 cosmid containing ROP5. Probed with α-rabbit ROP5 (IRDye 680: red) and mAb DG52 to SAG1 (IRDye 800: green–control), Stds: protein standards. The separate panels of the image come from the same gel where the intervening lanes have been removed as they were not related. (D) CD-1 mice were injected i.p. with indicated number of parasites, five mice per clone. The percentage of surviving mice was adjusted for those that were not infected, based on serological testing.

Because the peak on XII includes the tandem repeat of the *ROP5* gene that has been shown to be a virulence factor in other strains, we chose to investigate ROP5 further. To delete the large locus spanning all copies of *ROP5* on chromosome XII, we modified the *T*. *gondii* CRISPR-Cas9 plasmid [30] to express two separate single-guide RNAs (sgRNAs). The two sgRNAs target unique sites upstream and downstream of the *ROP5* locus (Fig 2A). When expressed in *T*. *gondii*, the sgRNAs should generate two double-strand breaks in the genome and allow for replacement of the intervening region with a selection cassette containing homologous regions outside the sgRNA cut sites. This strategy is expected to generate a knockout of all *ROP5* alleles including the expanded copy number *ROP5* genes represented by TGVAND_308090, and two adjacent single copy genes that encode predicted pseudokinases that are paralogs of ROP5: TGVAND_308093 and TGVAND_308096 (Fig 2A). We co-electroporated the double-CRISPR plasmid with a loxP-DHFR*-mCherry-loxP selection cassette into VAND and acquired stable parasites after selection with pyrimethamine. A PCR screen for integration of the loxP-DHFR*-mCherry-loxP selection cassette at the *ROP5* locus identified parasite clones that were positive for homologous integration (Fig 2B) and lacked all *ROP5* coding regions (S1A Fig). Western blot analysis with rabbit α-ROP5 confirmed that no protein expression was detectable (Fig 2C). Wild type VAND and two different clones of VANDΔ*rop5* parasites were injected i.p. into CD-1 mice to test virulence. Only mice infected with wild type parasites succumbed to infection. Mice infected with VANDΔ*rop5* survived the course of the experiment, even at a high dose of 10^5^ parasites (Fig 2D). Surviving mice from these experiments (Fig 2D) were tested for the generation of *T*. *gondii* antibodies via ELISA to ensure they were initially infected (S2 Fig). To confirm that ROP5 is sufficient for virulence we complemented the VANDΔ*rop5* parasite with the TOXOM52 cosmid that spans the _308090 tandem ROP5 locus, but does not include the adjacent _308093 or _308096 genes. TOXOM52 transgenic parasites were positive by PCR for the *ROP5* coding sequence (S1A Fig) and showed wild type levels of ROP5 protein expression (Fig 2C). Mice infected with VANDΔ*rop5*::TOXOM52 succumbed to infection in a similar timeframe as those infected with wild type parasites, demonstrating that the tandemly repeated _308090 *ROP5* alleles are sufficient to restore virulence to wild type levels (Fig 2D). These results confirm that *ROP5* is the major locus responsible for virulence differences between type 2 ME49 and type 10 VAND.

### ROP5 is a major virulence factor in Clade B South American strains TgCtBr5 and TgCtBr18

**Fig 3:**
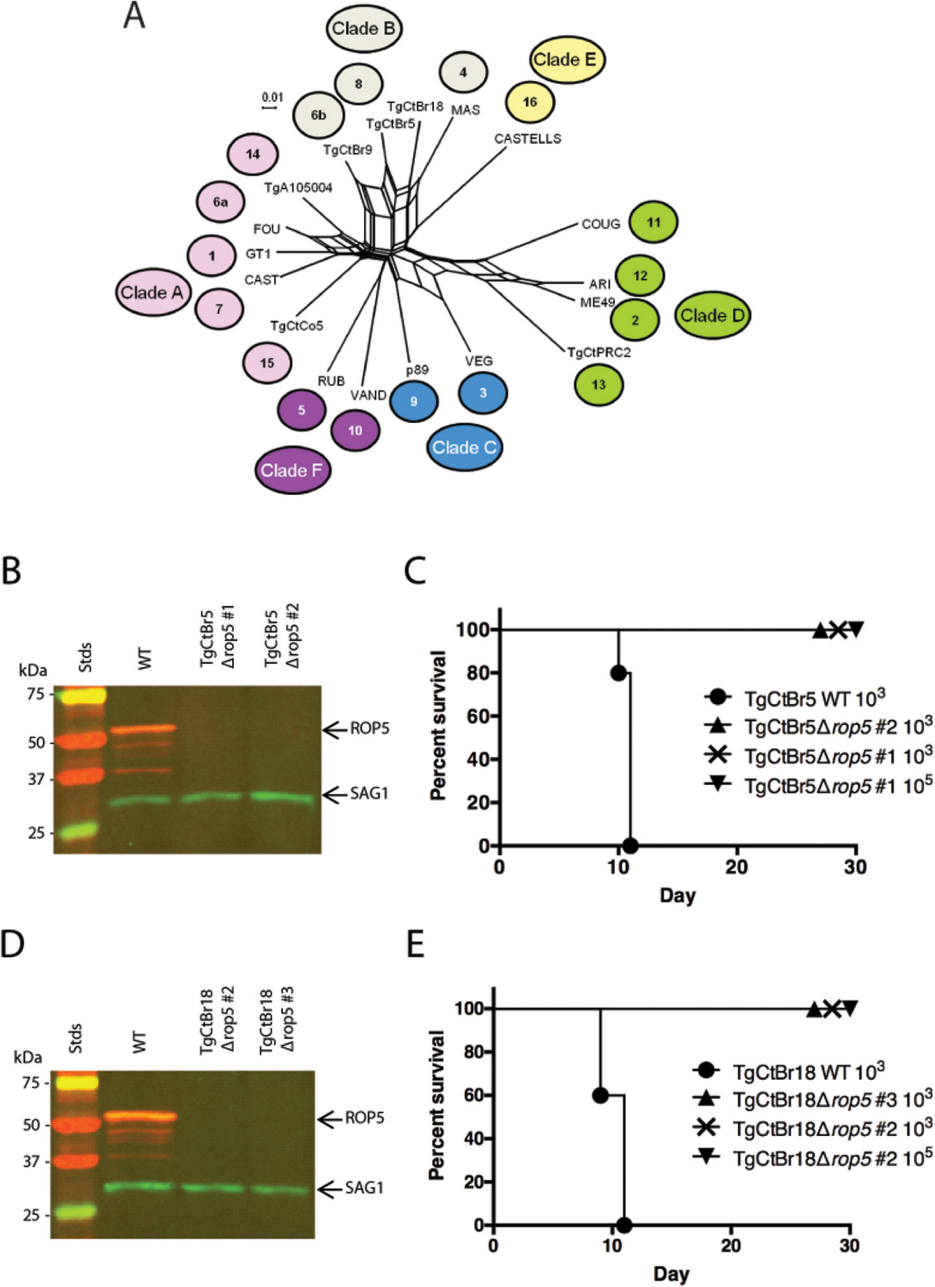
ROP5 is a virulence factor in South American strains TgCtBr5 and TgCtBr18. (A) Population genetic network analysis using genome-wide SNPs from 16 *Toxoplasma gondii* reference strains plus strain TgCtBr18. Numbers represent the haplogroup of the strain and colors represent the Clade: Clade A (pink), Clade B (gray), Clade C (blue), Clade D (green), Clade E (yellow), Clade F (purple). (B) Western blot of the TgCtBr5Δ*rop5* clones probing with rabbit α-ROP5 (IRDye 680: red) and mAb DG52 against SAG1 (IRDye 800: green–control), Stds: protein standards. (C) CD-1 mice were i.p. injected with indicated number of parasites, five mice per clone. (D) Western blot of the TgCtBr18Δ*rop5* clones probing with rabbit α-ROP5 (IRDye 680: red) and mAb DG52 against SAG1 (IRDye 800: green–control), Stds: protein standards. (E) CD-1 mice were i.p. injected with indicated number of parasites, five mice per clone. The percentage of surviving mice was adjusted for those that were not infected, based on serological testing.

Genetic crosses have now implicated ROP5 as a major virulence factor using type 1 GT1 [9] (Clade A), type 2 ME49 (Clade D), type 3 VEG (Clade C) [7,10] and type 10 VAND (Clade F) parasites as parental strains. These strain types represent a broad, yet incomplete, spectrum of the global *T*. *gondii* genetic diversity (Fig 3A) [21,22]. Given this, we were interested to test the contribution of ROP5 to virulence in strains from Clade B, a genetically distinct group not represented by the currently available genetic crosses. Using the double-CRISPR strategy applied to generate the VANDΔ*rop5* strain, we created Δ*rop5* parasites for virulent South American type 8 TgCtBr5 and type 4 TgCtBr18 strains. Knockouts were confirmed by PCR screening of clones for integration of the selection cassette (S3A and S3B Fig), loss of the *ROP5* coding region (S1B and S1C Fig), and loss of ROP5 protein expression by Western blotting (Fig 3B and 3D). Mice succumbed to infection with wild type strains of TgCtBr5 or TgCtBr18, yet when mice were infected with either TgCtBr5Δ*rop5* or TgCtBr18Δ*rop5* they survived the course of the experiment (Fig 3C and 3E). These data demonstrate that ROP5 is also a major contributor to virulence in Clade B strains of *T*. *gondii*.

### Loss of ROP18 renders South American strains VAND, TgCtBr5, and TgCtBr18 avirulent

**Fig 4:**
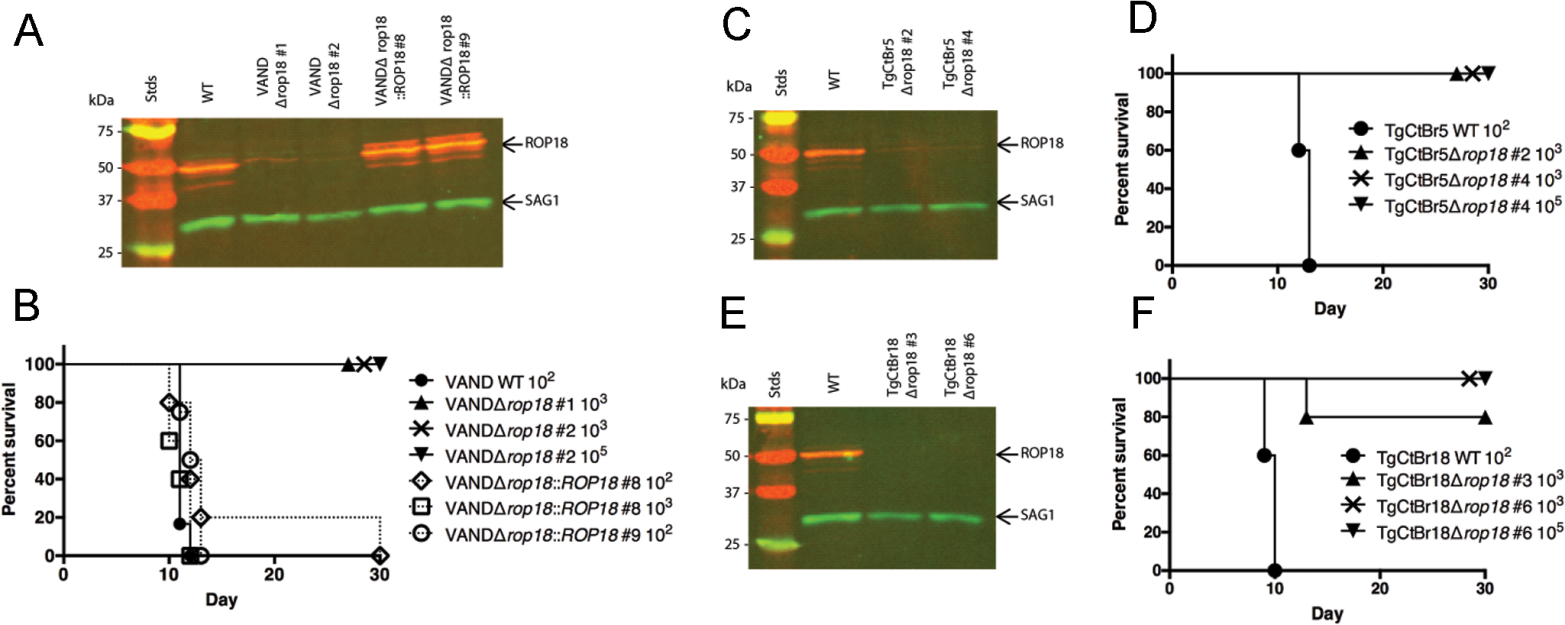
ROP18 is essential for virulence in South American strains VAND, TgCtBr5, and TgCtBr18. (A) Western blot of the VANDΔ*rop18* and complemented lines probed with rabbit α-ROP18 (IRDye 680: red) and mAb DG52 against SAG1 (IRDye 800: green–control), Stds: protein standards. (B) CD-1 mice were injected i.p. with indicated number of parasites, five mice per clone. (C) Western blot of the TgCtBr5Δ*rop18* clones probing with rabbit α-ROP18 (IRDye 680: red) and mAb DG52 against SAG1 (IRDye 800: green–control), Stds: protein standards. (D) CD-1 mice were injected i.p. with indicated number of parasites, five mice per clone. (E) Western blot of the TgCtBr18Δ*rop18* clones probing with rabbit α-ROP18 (IRDye 680: red) and mAb DG52 against SAG1 (IRDye 800: green–control), Stds: protein standards. (F) CD-1 mice were injected i.p. with indicated number of parasites, five mice per clone. The percentage of surviving mice was adjusted for those that were not infected, based on serological testing.

ROP5 and ROP18 have previously been shown to function together to resist host IRG-mediated parasite killing [16,18]. Although *ROP5* plays a dominant role in many strains, the effect of deleting *ROP18* varies with different backgrounds. For example, deletion of *ROP18* results in a delay in the time until death in the RH*Δrop18* strain [12] and a modest shift in the LD_50_ in GT1Δ*rop18* strain [30] parasites. Consequently, we assessed the role of *ROP18* in the virulence of South American VAND strains. Using the sgRNA-ROP18 CRISPR disruption strategy described previously [30], we deleted *ROP18* from the VAND-SNF^r^ parental strain. PCR screening for integration of the DHFR* selection cassette at the *ROP18* locus was confirmed in stable clones (S4 Fig), resulting in the loss of ROP18 protein expression, as determined by Western blotting (Fig 4A). Unlike the delayed death phenotype seen previously for type 1 RH*Δrop18* parasites, we observed 100% survival rates for CD-1 mice infected with two different clones of VANDΔ*rop18* parasites (Fig 4B), even at a dose of 10^5^ parasites. Virulence was restored in VANDΔ*rop18* (Fig 4B) when it was complemented with a ROP18-Ty tagged version of the protein (Fig 4A).

To determine how the loss of *ROP18* would affect virulence in Clade B strains, we knocked out *ROP18* in TgCtBr5 and TgCtBr18 using the same strategy used in VAND. After pyrimethamine selection we obtained parasite clones that integrated the DHFR* selection cassette at the *ROP18* locus (S4B and S4C Fig), resulting in the loss of expression of ROP18 as determined by Western blotting (Fig 4C and 4E). Similar to type 10 VAND parasites lacking ROP18, TgCtBr5Δ*rop18* and TgCtBr18Δ*rop18* parasites were completely avirulent in CD-1 mice, even at a dose of 10^5^ (Fig 4D and 4F)

### CNV and allelic structure of ROP5 in South American strains

**Fig 5:**
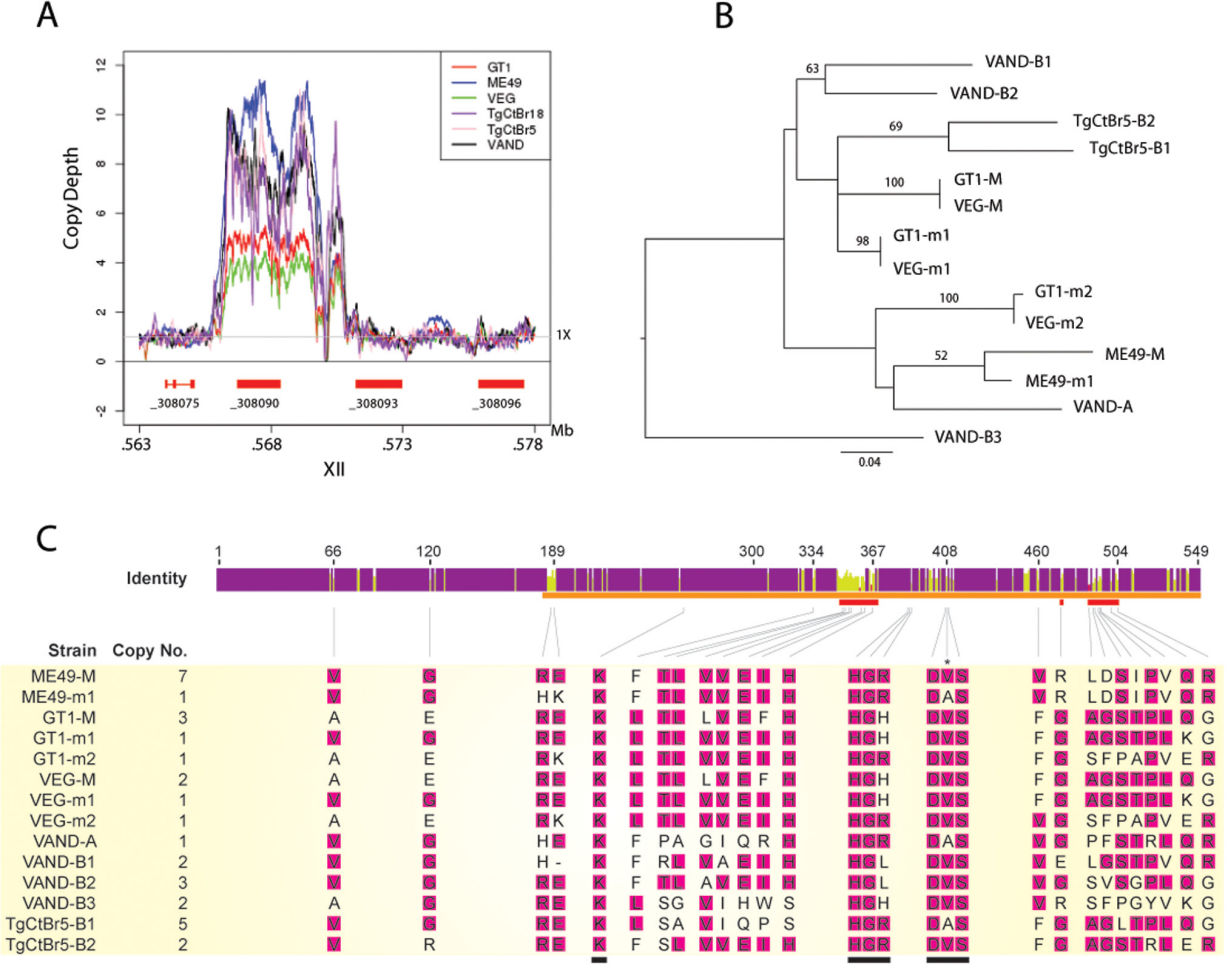
CNV and phylogenetic analysis of *ROP5* copies in VAND, TgCtBr5, TgCtBr18. (A) Copy number variation depth for *T*, *gondii* strains across the ROP5 locus on chromosome XII. Copy number estimates are based on the read depth per base pair normalized to 1X across the respective genome. (B) A Maximum Likelihood tree for *T*. *gondii* strains based on ROP5 amino acid sequences. Only maximum likelihood bootstrap values above 50 are shown. (C) The sequence identity across all sequences at each position is shown for the ROP5 amino acid sequence alignment. Purple indicates residues at positions with 100% sequence identity. Yellow indicates less than complete sequence identity and red indicates very low sequence identity at a given position. The ROP5 pseudokinase domain, catalytic triad, and residues involved in IRGa6 interactions are indicated by orange, black, and red bars, respectively. The predicted ROP5 allele copy numbers are shown beneath the sequence identity graph along with the amino acid alignments at positions where the respective codons are evolving under positive selection. The most frequent amino acid residue in a given position of the alignment is highlighted in pink. Amino acid positions/alignments of the catalytic triad are also shown; only a single codon position within the catalytic triad is evolving under positive selection (indicated by an asterisk). Allele names for the respective strains used in B and C are based on NCBI entries. Further discussion of the relationship among the alleles is provided in the results.

The locus encoding ROP5 shows evidence of expansion of tandem copies of the gene in different strains of *T*. *gondii* [10,16]. Alignments of genome-wide sequence to the assembled ME49 chromosomes revealed that the copy number for the *ROP5* gene (_308090) varies between strains, whereas the more divergent _308093 and _308096 ROP5 paralogs that lie adjacent in the genome are each single copy (Fig 5A) [31]. The number of copies of *ROP5* (_308090) (i.e. copy number variation (CNV)) for each of the strains was estimated by comparing the trace reads to the CDS, yielding the following estimates: GT1 (5), ME49 (10), VEG (4), TgCtBr18 (6), TgCtBr5 (7), and VAND (8) (Fig 5A and 5B). Previous studies have shown that each strain contains a dominant allele that is expressed at higher levels based on CNV. Dominant alleles have been referred to as “C”, while less common alleles were labelled as “A” or “B”, in work reported by Resse et al., [10], while these two categories were called M for major (C alleles) and m for minor alleles (B and A alleles) by Behnke et al., [9]. ROP5 alleles in VAND and TgCtBr5 were also previously named using A and B alleles, based on how they grouped phylogenetically with a collection of various strains including the type 2 strain ME49 [18]. We were able to estimate the CNV for the individual ROP5 alleles from these alignments for the strains VAND and TgCtBr5 (Fig 5C). These genes can also be defined as major and minor alleles based on copy number: TgCtBr5-B1 corresponds to the major allele (M), while TgCtBr5-B2 is a minor allele (m1); VAND-B2 is the major allele (M); VAND-B1, VAND-B3, and VAND-A are minor alleles (m1-3, respectively).

Although the number of ROP5 copies found in a strain doesn’t correlate with virulence, amplification of specific alleles types is associated with virulence in the murine model [10,16]. For example, the major alleles shared by type 1 and 3 strains (GT1-M, VEG-M corresponding to ROP5C_I/III_) are associated with virulence, while those from type 2 (ME49-M or ROP5C_II_) are not [9,10]. To expand on this relationship, we conducted phylogenetic analysis using the previous sequences for ME49, GT1, VEG [16] and VAND, TgCtBr5 [18] ROP5 alleles that are available from NCBI. Phylogenetic analysis of the ROP5 protein sequences revealed a lack of monophyly among the three strain types (Fig 5B). However, most alleles from the virulent strains group together, with VAND-B1 (m1), VAND-B2 (M), TgCtBr5-B1 (M), and TgCtBr5-B2 (m1) being closer to GT1-M. This cluster also included the major type 3 allele in VEG (VEG-M), which is functionally similar to the major allele in type 1 strains (the avirulence of VEG is due to hypo-expression of ROP18 not a defect in ROP5). In contrast, GT1-m2, VEG-m2, and VAND-A from virulent parasites group with the avirulent ME49-M and ME49-m1 alleles (Fig 5B). VAND-B3 appeared as a more divergent allele occurring on a long branch at the base of the tree (Fig 5B).

**Fig 6:**
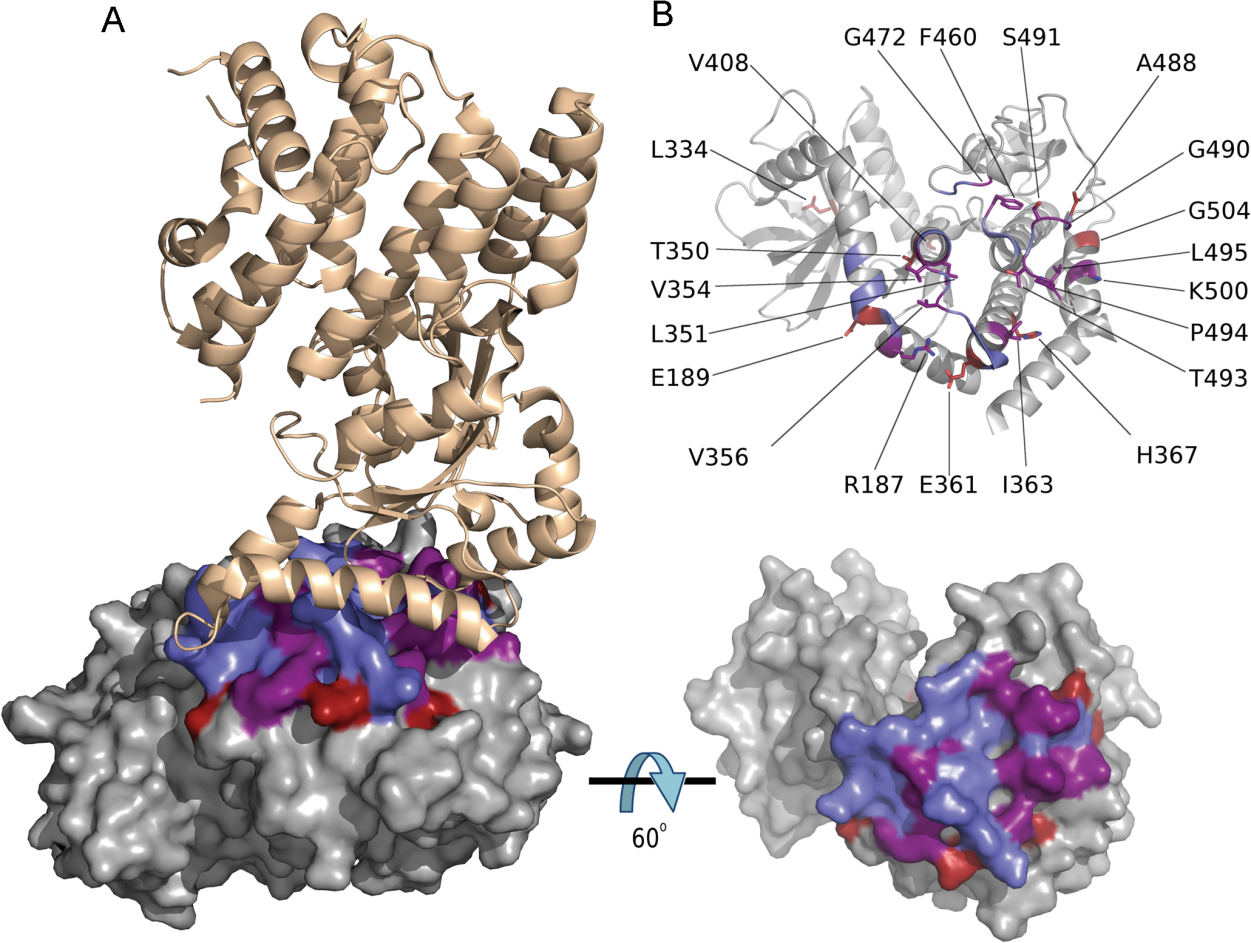
Codons under positive diversifying selection encode amino acid residues at the ROP5B_I_ /IRGa6 interface. (A) Structure of *T*. *gondii* ROP5B_I_ (grey) bound to murine Irga6 (tan) showing amino acid residues under positive diversifying selection (red), ROP5B_I_ residues at the ROP5B_I_ /Irga6 interface (blue), and residues that are under positive diversifying selection and at the interface (purple). (B) Both panels are a rotation of 60° about the x-axis with respect to A, showing ROP5B_I_ with Irga6 removed for clarity. The color scheme is as in A. Bottom panel is a surface representation showing the overlap in surface residues. The top panel shows the position of all amino acid residues under positive diversifying selection.

The function of ROP5 alleles in virulence may be attributed to differences the polymorphic surface of ROP5, which interacts with host IRGs and likely contributes to IRG specificity [19]. Indeed, when we analyzed the ratio of non-synonymous to synonymous mutations we detected 23 codon sites evolving under significant positive diversifying selection, with the majority of codon sites (74% of positive codons) localized within the regions encoding the polymorphic surface of ROP5 (S1 Table and Fig 5C). Interestingly, one codon site that evolves under positive diversifying selection was found within the DFG motif that is normally part of the activation loop in active kinases (S1 Table and Fig 5C). To provide greater insight into the region of ROP5 that is under diversifying selection, we mapped these residues on the recently described binding site for Irga6 as revealed by a X-ray co-crystal structure [19]. A majority of the residues in ROP5 that are under positive selection cluster tightly to a region that interacts directly with Irga6 (Fig 6A). A total of 13 residues (56.5%) under positive selection are directly at the interface between ROP5 and Irga6 (purple in Fig 6B, S1 Table), as identified by PDBePISA [32,33]. An additional 3 residues (13%) under positive selection are close to the interface and may play a supporting role in stabilizing the primary interface residues (S1 Table and Fig 6).

## Discussion

Our studies extend the application of forward genetic crosses and reverse genetic engineering using CRISPR-Cas9 to South American strains to provide an expanded framework for examining the basis of acute virulence in the mouse model. These studies reveal a conserved role for ROP5 and ROP18 in acute virulence in the mouse, likely due to their previously demonstrated role in protecting the parasite from host innate immune responses [12,15]. The identification of only a single significant QTL in analyzing phenotypic differences among progeny from the genetic cross between type 2 ME49 and type 10 VAND led to the identification of *ROP5* as the major locus in controlling acute virulence in the mouse. Although *ROP18* was not detected in a primary scan, a two-locus interaction occurred between regions containing *ROP5* (chromosome XII) and *ROP18* (chromosome VIIa), suggesting that these two genes collectively explain the major virulence differences among diverse strains of *T*. *gondii* in the mouse. This prediction was borne out by reverse genetic engineering of *ROP5* and *ROP18* disruptant mutants in other South American strains, the latter of which showed more severe defects in acute virulence than previously observed in the type 1 background. The widespread importance of these two virulence factors suggests that they evolved before the global spread and divergence of the parasite, consistent with recent reports that functional alleles of ROP5 and ROP18 also exist in the close relative *Hammondia hammondi* [34], even though this parasite is not virulent in mice. Collectively, these data suggest that ROP5 and ROP18 have been important in the evolution of these parasites within their rodent hosts, likely due to their interaction with the IRG family.

Despite extensive polymorphism between alleles, the major difference in the contribution of ROP18 to mouse virulence is due to different expression levels, being high in types 1 and 2, and very low in type 3 [7,8]. Complementation of a type 3 strain by over-expression of either ROP18_I_ [8] or ROP18_II_ [7] was sufficient to restore virulence. Additionally previous studies have shown that complementation of an avirulent type 3 strain with type 1-like alleles of ROP18 from South American strains results in a similar gain of virulence [24]. Combined with the fact that ROP18 was not seen as a major QTL in the cross between type 1 and 2, where ROP5 was mapped [9], these data suggest that different alleles of ROP18 function similarly, and their differential contribution to virulence is primary due to expression level. Given these previous findings, it is perhaps not surprising that *ROP18* was not identified as a major QTL in the present cross. However, it was somewhat surprising that *ROP5* was identified as the gene responsible for the majority of difference in acute virulence between type 2 ME49 and type 10 VAND, given the extensive genetic differences between these strains [21,22]. The mechanism by which ROP18 and ROP5 thwart innate immunity has been well established in North American strain types, where the combination of alleles present in type 1 strains leads to resistance to IRG loading relative to type 2 or 3 strains [11]. Although we did not test the recruitment of IRGs directly in this study, the role of ROP5 and ROP18 in the virulence of South American strains is likely due to their function in resisting host IRG recruitment to the parasitophorous vacuole. Consistent with this prediction, previous studies have shown that in IFNg-activated host cells, vacuoles containing virulent VAND (type 10) and TgCtBr5 (type 8) parasites recruit less Irgb6 than susceptible type 2 and 3 parasites [18].

The only other single peak observed on chromosome Ia was below the threshold of significance. In a previous genetic cross between type 1 and 3, a minor QTL was detected on chromosome Ia from markers M48 (~330 kbp)–AK4 (~485 kbp) with a LOD score of 2.38 [8]. However, in the present study, the QTL peak on chromosome Ia lies at marker MV24 near the right end of the chromosome (~1,500 kbp) with a LOD score of 2.54, and a marginal level of significance. There is also a second non-significant peak in the center of the chromosome from MV2 (~570 kbp) to MV9 (~830 kbp). These findings suggest that genes on this chromosome play a minor role in acute virulence in the mouse, although the resolution of the present data is insufficient to precisely map these or to identify candidate genes.

Although forward genetic mapping allowed us to identify a locus containing *ROP5* as important in the virulence of South American strains, the newly adapted CRISPR-Cas9 technology was critical for testing its function by reverse genetics. The default DNA damage repair pathway in *T*. *gondii* is nonhomologous DNA end joining, and for this reason the use of a Δ*ku80* background is important to efficiently generate knockout parasites [35,36]. Several attempts were made to generate a VANDΔ*ku80* strain for use in this study but all were unsuccessful, perhaps because VAND is a low passage isolate that has not been adapted to *in vitro* culture conditions for long periods of time. However, gene disruption using CRISPR-Cas9 is highly efficient even in wild type *KU80* proficient parasites, as shown previously [30], and this technique was also successfully applied here to diverse strains. We also extended this strategy by creating a CRISPR-Cas9 plasmid that expressed two sgRNAs targeting sites surrounding a large genomic locus. This strategy was efficient at deleting the ~30 kb *ROP5* locus in three different low-passage South American strains. The acute virulence phenotype of VAND*Δrop5* mutant was restored using a cosmid that only contains the *ROP5* cluster, but not the adjacent pseudokinase paralogs, indicating that these adjacent genes contribute little to the phenotype and that the *ROP5* alleles are the primary determinants of acute virulence.

Phylogenetic analysis revealed that VAND and TgCtBr5 contain *ROP5* alleles that are similar to the major alleles in the virulent type 1 strain GT1, with only minor alleles being similar to the type 2 strain ME49. Based on this similarity, we decided to complement the VAND *ROP5* locus knockout with a cosmid containing the cluster from the type I RH strain. We have not tested the function of individual alleles in restoring this phenotype, nor complemented directly with individual VAND alleles, and further testing would be needed to establish if they have conserved functions. Interestingly, all of the ROP5 alleles contain a functional change in HRD motif, that forms part of the catalytic triad, where major alleles in the virulent strains (those related to GT1-M corresponding to ROP5C_I_) and some minor alleles such as GT1-m1 (ROP5B_I_) contain “H”, while the major allele in the type 2 strain ME49 (ME49-M, ROP5C_II_), and minor alleles such as VAND-A, GT1-m2 (ROP5A_I_), and VEG-m2 contain a “R”. In contrast, VAND-B1 and VAND-B2 alleles show a new residue in the catalytic triad of “L”, which again is predicted to lack kinase activity. None of these residues is predicted to be active as this residue is normally a D in active kinases [37]. The functional significance of these changes in the catalytic triad of ROP5 is unclear as the interface that interacts with IRGs is on the opposite face of the molecule [19]. However these different alleles also correlate with differences in function in laboratory mice: the former group, including GT1-M, GT1-m1, and related alleles, inhibit recruitment of IRGs to the parasitophorous vacuole *in vivo*, while the later group, including ME49-M and related alleles, do not [16,17]. The diversity of alleles found in ROP5 suggests that alleles that lack activity in the mouse may be active in others hosts, for example other rodents or birds.

Previous studies have emphasized that this binding interface is polymorphic in ROP5 [19], and our analysis of residues that are under strong positive selection in ROP5 further defines this interface as a cluster of residues that directly interacts with Irga6. Additional nearby polymorphic residues in ROP5 may act to influence the Irga6 interaction through allosteric interactions, or they may be important in binding to other polymorphic alleles of Irga6. Importantly, IRGs also show polymorphism in this region of the molecule [19], suggesting that ROP5 is under selective pressure for enhanced binding while IRGs have evolved to avoid recognition. In support of this model, binding of ROP5 to Irga6 has been shown to directly limit polymerization *in vitro* [19], as well as to enhance phosphorylation of IRGs by ROP18 *in vitro* and *in vivo* [16]. Collectively, these findings provide evidence for the hypothesis that the polymorphic regions of ROP5 are evolving under positive diversifying selection driven by interaction with host innate immunity factors.

We were also able to efficiently delete *ROP18* in different genetic backgrounds, demonstrating the potential of CRISPR-Cas9 for genetic manipulation of diverse strains of *T*. *gondii*. Unlike *ROP5* knockouts where all Δ*rop5* strains tested are equally avirulent despite different genetic backgrounds, there are large differences in the phenotype of *Δrop18* disruptants between strains. For example, previous studies have reported that deletion of *ROP18* in the type 1 RH strain led to delayed time until death, but not a change in the lethal dose [12] vs. a several log increase in the lethal dose using the type 1 GT1 strain [30]. In contrast, deletion of *ROP18* in type 4, 8, and 10 strains led to a complete loss of virulence with no animals dying at a challenge dose of up to 10^5^ (present study). The reasons for these differences are uncertain but are unlikely to be due to experimental variation in the mouse model since they were obtained in the same laboratory using a standard protocol that is highly reproducible. Type 1 strains normally result in lethal infection in all strains of conventional laboratory mice with an inoculum of a single parasite [4,5,6]. In contrast animals infected here with Δ*rop18* mutants in type 4, 8, and 10 did not succumb to infection, yet had very high antibody titers, reflecting an active infection. Previous studies have stressed that the major contribution of ROP18 in acute virulence appears to be due to expression level differences, and yet all the strains tested here show high levels of expression [24]. Hence, it is not immediately clear why the contribution of ROP18 to acute virulence would vary so extensively among the strains tested here. Once possibility is that the small number of polymorphisms in *ROP18* alleles found between type 1 strains and the isolates studies here may account for these differences. Consistent with this, previous studies have also shown that virulent alleles of ROP18 are also under diversifying selection [24]. The marked differences in attenuation with the deletion of ROP18 might also be due to the relative importance of other genes that contribute to acute virulence. For example, the active rhoptry kinase ROP17 [20] or the secreted dense granule GRA7 [38], which have been shown to be important in the type I RH strain, may partially compensate for loss of ROP18 in some backgrounds. The fact that ROP18 plays a less important role in the RH strain, may also be due to its passage history, which has been propagated extensively in mice and *in vitro* since its original isolation more than 75 years ago [39], resulting in numerous changes that favor growth. Further studies of the precise function of various *ROP18* alleles in different genetic backgrounds would be necessary to decipher among these alternatives.

Despite their conserved functions in the murine model, ROP5 and ROP18 do not appear to mediate resistance to IFNg-activated control of replication in human cells [18]. Because humans lack the expanded IRG family [40], other mechanisms of control likely contribute to cell autonomous control of replication. However, ROP18 has also been shown to phosphorylate host cellular protein ATF6-β targeting it for degradation, thus hindering antigen presentation [41]. This pathway may contribute to disruption of adaptive immunity in hosts other than murine, including humans. Consistent with this possibility, among human patients with ocular toxoplasmosis in Columbia, mouse-virulent alleles of ROP18 were correlated with more severe disease [42]. Additionally, certain South American strains appear to be more virulent in humans in that they are associated with higher rates of ocular disease in the case of Clade B [43], and they are associated with severe disease in healthy individuals in the case of Clade F [44,45,46]. The parasite factors responsible for increased acute disease severity in humans are unknown, but these traits could be suitable for genetic mapping if appropriate screens were available for assessing these phenotypes *in vitro*. Although it is unlikely that resistance in human cells is a driving factor for evolution of *T*. *gondii* virulence, given the fact that humans are rarely involved in transmission, such traits could represent conserved functions that are important in multiple vertebrate hosts.

Using forward genetic mapping and reverse genetic engineering with CRISPR-Cas9, we demonstrate the conserved nature of the ROP5 and ROP18 virulence factors in South American strains of *T*. *gondii*. We also found a more dramatic role for ROP18 in the virulence of these strains than had been appreciated using North American isolates of the parasite. Recent genetic studies in the house mouse reveal novel alleles of IRGs that are responsible for their enhanced resistance to otherwise virulent type 1 lineages of *T*. *gondii* [47]. The predator-prey relationship between the house mouse and domestic cat has likely existed for at least 10,000 years in association with human settlement [48]. In contrast, strains harboring similar ROP18 and ROP5 alleles have been estimated to have diverged several million years ago [22]. Collectively these studies suggest that parasite virulence determinants are co-evolving against the pressure of murine immunity factors in a backdrop of more ancient history that is conserved among diverse strains of *T*. *gondii*.

## Materials and Methods

### Ethics statement

All animal experiments were conducted according to the U.S.A. Public Health Service Policy on Humane Care and Use of Laboratory Animals. Animals were maintained in an Association for Assessment and Accreditation of Laboratory Animal Care International-approved facility. All protocols were approved by the Institutional Care Committee at the School of Medicine, Washington University in St. Louis.

### Parasite culture

The parents and progeny of the genetic cross between type 2 ME49-FUDR^r^ and type 10 VAND-SNF^r^ strains [25], and parasite strains TgCtBr5-SNF^r^ and TgCtBr18-SNF^r^ were grown as tachyzoites in human foreskin fibroblast (HFF) monolayers. The TgCtBr5 and TgCtBr18 strains were made SNF resistant using *N*-ethyl-*N*-nitrosourea as described previously [49]. Cultures were maintained in D10 media composed of Dulbecoc’s modified Eagle medium (DMEM), 10% fetal bovine serum (FBS), gentamycin, and glutamine at 37°C with 5% CO_2_. Infected monolayers were scrapped and force passaged through 21 gauge needles to harvest parasites.

### Acute infection and ELISA

CD-1 mice were acquired from commercial vendors (Jackson Labs) and housed under SPF conditions at Washington University School of Medicine. Acute virulence was determined by i.p. injection of indicated number of tachyzoites into groups of five 8–12 week old female outbred CD-1 mice per experiment. Survival was monitored for 30 days, after which surviving mice were bled to collect serum for serological testing by enzyme-linked immunosorbent assay (ELISA), as described previously [50]. The percentage of surviving animals was determined by the number of animals that succumbed / total number of animals that were infected x 100. Survival values were corrected for animals that failed to become infected (i.e. those that remained serologically negative).

### Quantitative trait locus analysis for virulence

The virulence phenotype was analyzed by QTL mapping using J/qtl [51] to establish the genome-wide association of phenotypes with genotypes of progeny from the genetic cross between type 2 ME49-FUDR^r^ and type 10 VAND-SNF^r^ strains described previously [25]. Virulence QTLs were confirmed by interval mapping based on a one-dimensional genome scan with 1,000 permutation to generate a log likelihood plot (*P* < 0.001). Covariant analysis and a two-dimensional genome scan were conducted using J/qtl to identify minor QTLs and epistatic interactions, respectively using criteria above.

### Double-CRISPR and pUC19-ROP5HR-DHFR*mCherry plasmids

In order to make the double-CRISPR plasmid used to knockout the *ROP5* locus, two single sgRNA CRISPR plasmids were constructed. The pSAG1::CAS9-U6::sgUPRT plasmid (Addgene #54467) was used as a template to amplify with Set1-1 or Set1-2 primers (S2 Table) and PCR products were processed using the Q5 Site-Directed Mutagenesis Kit (NEB), as described previously [30] to create the pSAG1::CAS9-U6::sg5’ROP5 and pSAG1::CAS9-U6::sg3’ROP5 plasmids. The sgRNA cassette (containing the U6 promoter, sgRNA, and scaffold) was PCR amplified with Q5 High-Fidelity DNA polymerase (NEB) from the pSAG1::CAS9-U6::sg3’ROP5 plasmid using Set2-1 primers (S2 Table) that contain terminal KpnI and XhoI restriction enzyme sites. The sg3’ROP5 cassette PCR product and the pSAG1::CAS9-U6::sg5’ROP5 plasmid were cut with KpnI/XhoI (NEB), buffer 4 at 37°C overnight, gel purified on a 1% agarose gel, and purified with the Qiagen Gel Purification Kit (Qiagen). The digested and purified PCR products and plasmid were ligated using T4 ligase (NEB) at 16°C over-night, ligations were transformed into OmniMAX chemically competent cells (Invitrogen), and a correct clone was identified with KpnI/XhoI restriction fragment mapping. The plasmid named pSAG1::CAS9-U6::sg5’ROP5-sg3’ROP5 plasmid contains two sgRNAs targeting the 5’ and 3’ side of the ROP5 locus.

To select for the excision of the *ROP5* locus in pSAG1::CAS9-U6::sg5’ROP5-sg3’ROP5 transfected parasites, we constructed a selection cassette flanked by homologous regions (HR) adjacent to the 5’ and 3’ sg*ROP5* CRISPR cut sites using Gibson Assembly (NEB). Four PCR fragments were amplified using Q5 High-Fidelity DNA polymerase (NEB) from the following template/primer pairs: (1) the p-loxP-DHFR*-mCherry-loxP plasmid with Set3-1 primers, (2) VAND genomic lysate with Set 3-2primers, (3) VAND genomic lysate with Set3-3 primers, (4) the pUC19 plasmid with Set3-4 primers (S2 Table). PCR products were gel purified as above and combined in a Gibson Assembly reaction (NEB) using manufactures protocol. The reaction was transformed into NEB 5-alpha cells and a correct clone was identified using three different restriction fragment mappings (BamHI/NotI, BamHI/ScaI, and ScaI/NotI) and named pUC19-*ROP5*HRs-loxP-DHFR*-mCherry. This plasmid was used as a template in a Q5 High-Fidelity DNA polymerase PCR to amplify the 5’*ROP5*HR-loxP-DHFR*mCherry-loxP-3’*ROP5*HR cassette using the Set4-1 primers (S2 Table). This amplified cassette was gel purified as above for use as a selection cassette in creating *ROP5* KO parasites.

### *ROP5* KOs and complementation

The VAND-SNF^r^, TgCtBr5-SNF^r^, and TgCtBr18-SNF^r^ strains were individually electroporated with the double-CRISPR pSAG1::CAS9-U6::sg5’ROP5-sg3’ROP5 plasmid and a PCR amplified 5’*ROP5*HR-loxP-DHFR*mCherry-loxP-3’*ROP5*HR selection cassette. Parasites were selected in 2 μM pyrimethamine (Sigma-Aldrich), cloned, and screened by PCR for integration of the DHFR*-mCherry cassette at the ROP5 locus using Set5-1 primers for 5’ integration, Set5-2 primers for 3’ integration, Set5-3 for the ROP5 coding sequence and Set5-4 primers to the *GRA1* promoter as a positive control for the PCR (S2 Table). Knockout parasites were identified, protein harvested, and Western blotted to confirm the loss of ROP5 protein.

In order to complement with a cosmid containing the DHFR* pyrimethamine selection cassette, we excised loxP-DHFR*-mCherry-loxP from a VAND *ROP5* knockout clone using Cre recombinase. The VANDΔ*rop5* #10 strain was electroporated with the pmin-Cre-eGFP plasmid [52], cloned without selection, and mCherry negative parasites were screened by immune-fluorescence assay (IFA) using primary rat aντι-mCherry mAb (Life Technologies) and secondary goat aντι-rat Alexa Fluor 594 (Life Technologies), to identify a VANDΔ*rop5*Δdhfr*mCherry parasite. Sensitivity to 2 μM pyrimethamine was confirmed for VANDΔ*rop5*Δdhfr*mCherry. To generate the complement, the VANDΔ*rop5*Δdhfr*mCherry parasite was electroporated with the TOXOM52 cosmid, derived from the type I RH strain (http://toxomap.wustl.edu/cosmid.html) [53]. The parasites were selected with 2 μM pyrimethamine (Sigma-Aldrich), and clones were screened for expression of ROP5 by IFA using primary rabbit anti-ROP5 and secondary goat anti-rabbit Alexa-Fluor 594 (Life Technologies). ROP5 expressing clones were identified, protein harvested, and Western blotted to confirm wild type levels of expression in the complement, creating VANDΔ*rop5*::ROP5.

### *ROP18* KOs and complementation

The *ROP18* KOs and complement were created as described previously [30]. Briefly, the VAND-SNF^r^, TgCtBr5-SNF^r^, and TgCtBr18-SNF^r^ strains were individually electroporated with the pSAG1::CAS9-U6::sgROP18 CRISPR plasmid and a PCR amplified selection cassette containing *ROP18* homologous regions flanking a DHFR* pyrimethamine resistance marker. Parasites were selected with 2 μM pyrimethamine (Sigma-Aldrich), cloned by limiting dilution, and PCR screened for integration of the selection cassette at the *ROP18* CRISPR cut site. Knockout clones were identified, proteins harvested, and Western blots were run to confirm the loss of ROP18 protein. To create a complemented strain, the VANDΔ*rop18* #1 clone was electroporated with the pSAG1::CAS9-U6::sgUPRT plasmid and a PCR amplified selection cassette containing UPRT homologous regions flanking an IMC1p-ROP18Ty expression construct. Parasites were selected with 10 μM FUDR (Sigma-Aldrich), cloned and screened for expression of Ty by IFA: using primary α-Ty-BB2 mAb and secondary goat-α-mouse Alexa Fluor 488 (Life Technologies). ROP18-Ty expressing clones were identified and protein harvested, and Western blots were run to confirm wild type expression levels of ROP18 in the complement, VANDΔ*rop18*::ROP18.

### Genomic lysate/PCR screens

Genomic lysates were harvested from various parasite strains for use as templates in PCR screens. Parasites were grown in HFF monolayers, harvest by scrape/needle pass, spun at 400 rcf and supernatant was aspirated from the parasite pellet. The pellet was suspended in genomic lysate buffer (1X PBS with 10% Taq polymerase buffer (Invitrogen) and 400 ng/μl proteinase K (Life Technologies)), the sample was incubated at 37°C for 1hr, 50°C for 30 min, and 95°C for 5–10 min, and spun to pellet debris.

Genomic lysates were used in PCR screens to detect the integration or removal of selection cassettes. For the ROP5 knockouts and complementation the primer sets outlined in S2 Table were used, and for the ROP18 disruptants and complementation the primer sets described previously [30] were used in a Taq polymerase (NEB) PCR. PCR products were run on a 1% agarose gel and imaged on a UV light box.

### Protein harvest/western blotting

Parasites were grown and harvested as above, counted on a hemocytometer, the pellet was resuspended in protein lysate buffer (1X PBS, 1% Triton-X, 1% DNase, 1% protease inhibitors (Sigma-Aldrich)) to 1 X 10^6^ parasites/μl, vortexed, and spun to pellet debris. Each sample contained protein from 5 X 10^6^ parasites in 1X sample buffer with 10% β-Me (Sigma-Aldrich) and was incubated for 5–10 min at 95°C. Samples were resolved on a 10% SDS-polyacrylamide (Bio-Rad) gel at 100V for 1 hr. Gels were transferred to nitrocellulose, blocked in 5% milk, and probed with (all diluted to 1:10,000) primary—rabbit α-ROP5 or rabbit α-ROP18 and mouse mAb DG52 α-SAG1 antibodies, and secondary antibodies consisting of goat α-rabbit IgG IRDye 680 (LI-COR Biosciences) and goat α-mouse IgG IRDye 800 (LI-COR Biosciences). All Blots were imaged on a LI-COR Odyessy CLx infrared imaging system (LI-COR Biosciences).

### Network analysis and CNV

Network analysis using genome wide SNP data from the 16 *Toxoplasma gondii* reference genomes and the TgCtBr18 genome (obtained from ToxoDB) was conducted using SplitsTree v4.4 [54] to generate unrooted phylogenetic networks using a neighbor-net method and 1,000 bootstrap replicates, as previously described [31].

ROP5 alleles used in this study were defined in previous studies [9,18] and were obtained from NCBI accession records for the following strains: GT1, VEG, and ME49 alleles (BK008043.1-BK008057.1), VAND alleles (JQ743716.1, JQ743730.1, JQ743760.1, JQ743772.1, JQ743778.1, JQ743783.1), TgCtBr5 alleles (JQ743734.1, JQ743742.1, JQ743743.1). Briefly, ROP5 alleles for GT1, VEG and ME49 were previously reconstructed in Behnke *et al*. [9] by aligning Sanger sequences of each strain to a reference ROP5 CDS allowing for the reconstruction of SNPs patterns. In this case, the Sanger sequences are long enough to combine overlapping reads containing the same SNP patterns together to define novel alleles, as detailed in Figure S6 of Behnke et al., [9]. Alleles for VAND and TgCtBr5 were previously defined by allele-specific amplification and direct sequencing as described in Niedelman *et al*. [18].

Copy-number variation (CNV) for the ROP5 locus was determined as previously described [31]. Briefly, NextGen sequence reads of the respective genomes were aligned to the ME49 assembled chromosomes using bowtie2 –end-to-end [55] the average 1X read bases for each genome and the average read bases for TGME49_308090, TGME49_308093, and TGME49_308096 were determined using samtools mpileup exported data [56]. The reads surrounding the *ROP5* locus for each genome were exported with samtools mpileup and data were graphed in R. To determine CNV for individual ROP5 alleles, the reads aligning to the region surrounding TGME49_308090 were exported from the bowtie2 alignment using mpileup and realigned to the ME49 assembled chromosomes using CLC Genomics Workbench. Reads that corresponded to a particular allele were identified based on SNP pattern and the allele CNV was estimating by calculating the ratio of allele reads as compared to the total CNV for the ROP5 locus [9].

### Phylogenetic analysis and diversifying selection

DNA sequences were translated and aligned using MUSCLE with Geneious v7.0.4. Phylogenetic analysis of the protein sequences was implemented in RAxML [57] using maximum likelihood and the JTT+I+G+F model. For maximum likelihood, we used the ModelGenerator v85 [58] to determine the most appropriate amino acid substitution model based on the Akaike Information Criterion. Positively selected codons were estimated by the Fast Unconstrained Bayesian AppRoximation method using HyPhy package [59,60]. Posterior probability values above 0.95 were chosen for codon sites where the distribution of non-synonymous substitution rates (β) are significantly greater than the synonymous (α) substitution rate.

### Molecular modeling studies

Amino acid residues at the ROP5/IRGa6 interface were identified by PDBePISA [32,33] based on the crystal structure of ROP5B_I_ (corresponding to ROP5 GT1-m1 in the present study) bound to murine IRGa6 (PDB code 4LV5) [19]. Residues close to the interface were identified by visual inspection. Figures were generated in PyMOL.

## Supporting Information

S1 Fig Diagnostic PCR for *ROP5* CDS. (A-C) Diagnostic PCR showing the absence of *ROP5* CDS in VAND (A), TgCtBr5 (B), and TgCtBr18 (C) ROP5 KO strains, and the restoration of *ROP5* in the VAND complemented strains. Set5 primers used, see S2 Table and Fig 2A. G = GRA1 promoter, 3 = *ROP5* CDS (Set5-3 primers).
(TIF)

S2 Fig ELISA on serum of surviving mice. Sera from all surviving mice in this study were tested for general Toxoplasma antibody in an ELISA using RH lysate. Positive control (green), negative controls (red), surviving mice (blue). Red line indicates 99% confidence level cut-off for calling positive samples.
(TIF)

S3 Fig Diagnostoc PCR for integration at *ROP5*. (A,B) Diagnostic PCR showing the integration of the DHFR*-mCherry cassette at the *ROP5* locus for TgCtBr5 (A) and TgCtBr18 (B) ROP5 KO strains. Set5 primers used, see S1 Table and Fig 2A.
(TIF)

S4 Fig Diagnostic PCR for integration at *ROP18*. (A-C) Diagnostic PCR showing the integration of the DHFR*-mCherry cassette at the *ROP18* locus for VAND (A), TgCtBr5 (B), and TgCtBr18 (C) ROP18 KO strains, as in *Bang et al*. *mBIO 2014*. Set6 primers used, see S2 Table.
(TIF)

S1 Table Codons under positive diversifying selection. (PDF)

S2 Table Primers used in this study. (PDF)
