# Supplementary figures and images for: Rhoptry Proteins ROP5 and ROP18 Are Major Murine Virulence Factors in Genetically Divergent South American Strains of Toxoplasma gondii
Source: PLoS Genet. 2015 Aug 20;11(8):e1005434. doi: 10.1371/journal.pgen.1005434 (PMC4546408; doi:10.1371/journal.pgen.1005434)

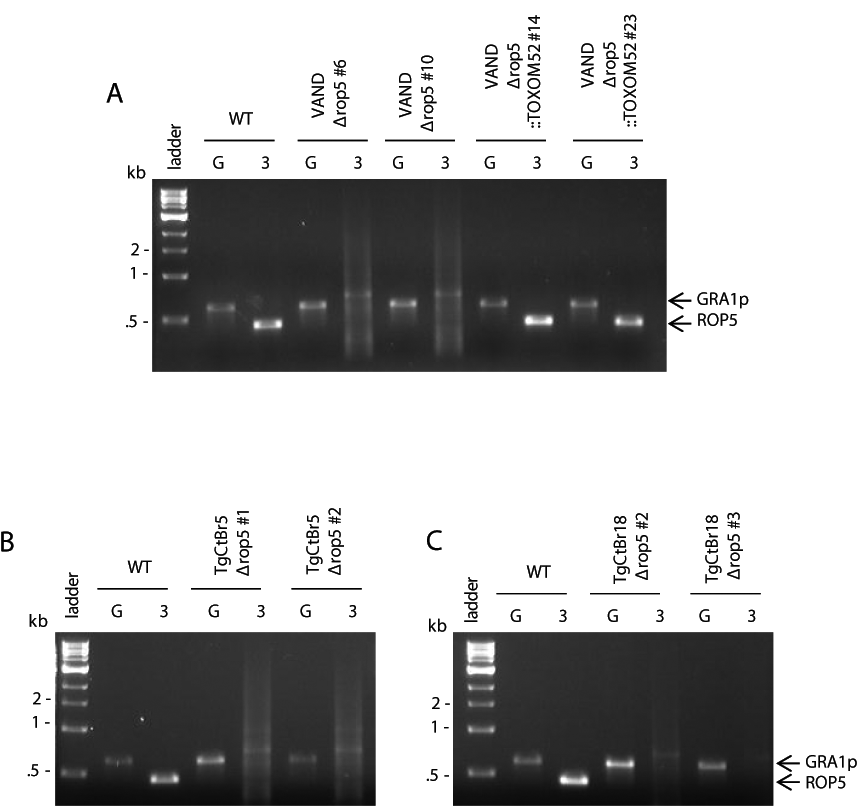

Supplement: S1 Fig — (A-C) Diagnostic PCR showing the absence of ROP5 CDS in VAND (A), TgCtBr5 (B), and TgCtBr18 (C) ROP5 KO strains, and the restoration of ROP5 in the VAND complemented strains. Set5 primers used, see S2 Table and Fig 2A. G = GRA1 promoter, 3 = ROP5 CDS (Set5-3 primers). (TIF) [file pgen.1005434.s001.tif]

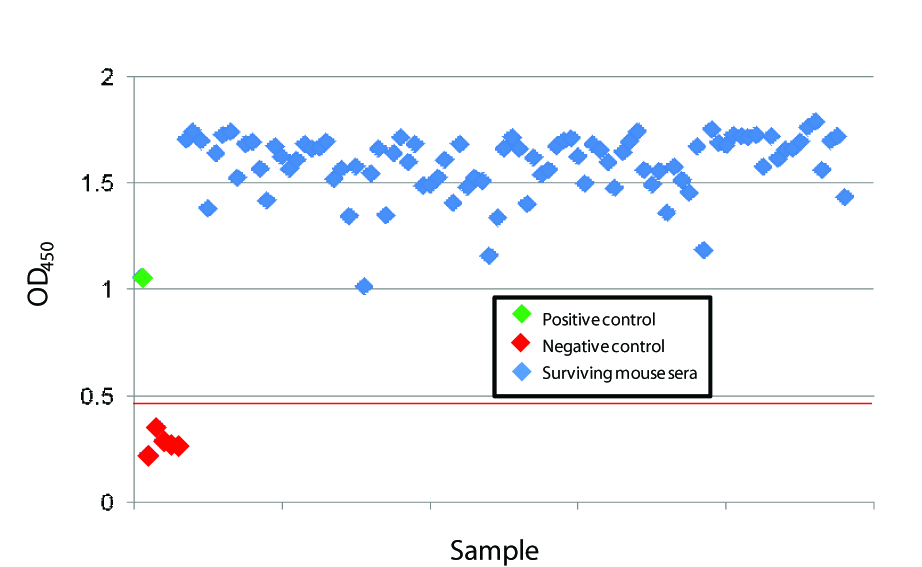

Supplement: S2 Fig — Sera from all surviving mice in this study were tested for general Toxoplasma antibody in an ELISA using RH lysate. Positive control (green), negative controls (red), surviving mice (blue). Red line indicates 99% confidence level cut-off for calling positive samples. (TIF) [file pgen.1005434.s002.tif]

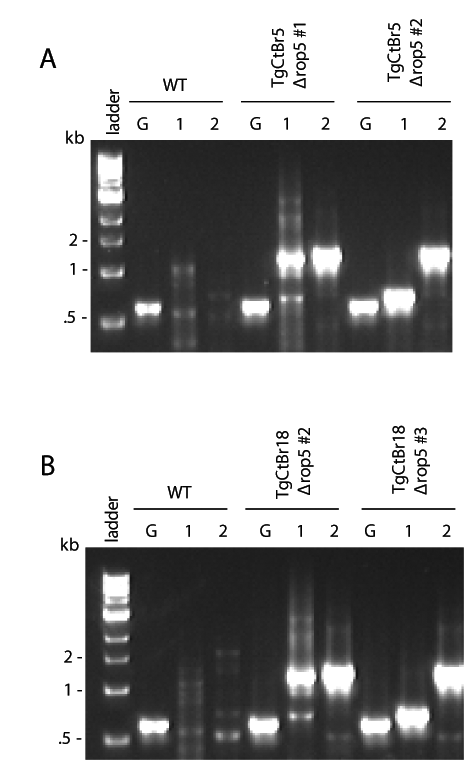

Supplement: S3 Fig — (A,B) Diagnostic PCR showing the integration of the DHFR*-mCherry cassette at the ROP5 locus for TgCtBr5 (A) and TgCtBr18 (B) ROP5 KO strains. Set5 primers used, see S1 Table and Fig 2A. (TIF) [file pgen.1005434.s003.tif]

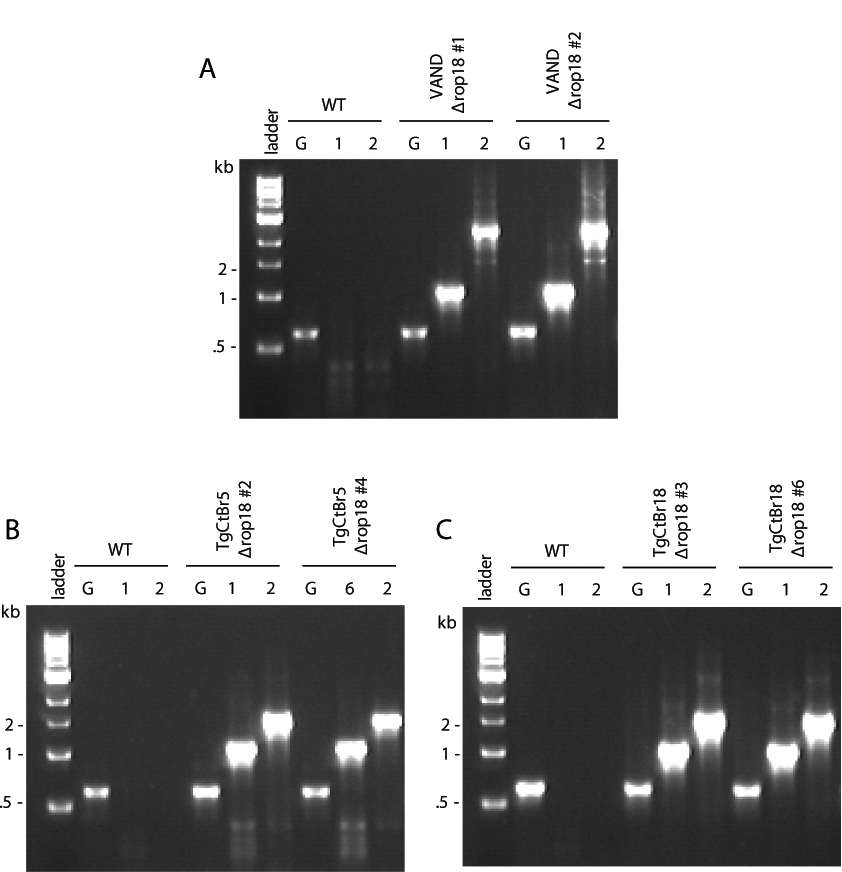

Supplement: S4 Fig — (A-C) Diagnostic PCR showing the integration of the DHFR*-mCherry cassette at the ROP18 locus for VAND (A), TgCtBr5 (B), and TgCtBr18 (C) ROP18 KO strains, as in Bang et al. mBIO 2014. Set6 primers used, see S2 Table. (TIF) [file pgen.1005434.s004.tif]
