# Supplementary material for: Rhoptry Proteins ROP5 and ROP18 Are Major Murine Virulence Factors in Genetically Divergent South American Strains of Toxoplasma gondii
Source: PLoS Genet. 2015 Aug 20;11(8):e1005434. doi: 10.1371/journal.pgen.1005434 (PMC4546408; doi:10.1371/journal.pgen.1005434)
Supplement: S1 Table — (PDF) [file pgen.1005434.s005.pdf]

S1 Table - Codons Under Positive Diversifying Selection

Codon	α	β	β-α	Posterior Prob β>α
66	0.301091	4.66745	4.36636	0.976829
120	0.350605	4.68078	4.33017	0.97017
187	0.323312	4.30613	3.98282	0.970214
189	0.488929	4.59146	4.10253	0.952346
334	0.515238	4.81452	4.29928	0.957139
350	0.326471	6.35042	6.02395	0.987242
351	0.384769	9.89544	9.51067	0.993081
354	0.340685	4.83471	4.49403	0.972795
356	0.344013	5.97709	5.63307	0.983814
361	0.528979	5.67777	5.14879	0.963
363	0.534201	11.2879	10.7537	0.988671
367	0.492919	4.89027	4.39735	0.960324
408	0.321548	4.89743	4.57588	0.975818
460	0.339681	4.47174	4.13206	0.969531
472	0.343514	4.7033	4.35978	0.971396
488	0.375668	12.4296	12.0539	0.996496
490	0.378753	12.1675	11.7888	0.996492
491	0.321108	5.0999	4.77879	0.977679
493	0.354172	8.83888	8.48471	0.992952
494	0.322563	5.9022	5.57963	0.985891
495	0.302877	5.68572	5.38285	0.983874
500	0.596215	5.83342	5.2372	0.957938
504	0.342009	4.8593	4.51729	0.973169
