# Supplementary material for: Rhoptry Proteins ROP5 and ROP18 Are Major Murine Virulence Factors in Genetically Divergent South American Strains of Toxoplasma gondii
Source: PLoS Genet. 2015 Aug 20;11(8):e1005434. doi: 10.1371/journal.pgen.1005434 (PMC4546408; doi:10.1371/journal.pgen.1005434)
Supplement: S2 Table — (PDF) [file pgen.1005434.s006.pdf]

S2 Table - Primers used in this study

	Primer Sequence
**ROP5 CRISPR plasmids - Set1**	
**pair1**	
ROP5 CRISPR 5' of locus - for (sgRNA-1)	5' GTA GTC GTG TGT ACA GTA TCG TTT TAG AGC TAG AAA TAG C 3'
Universal sgRNA plasmid primer - rev (sgRNA-2)	5' AAC TTG ACA TCC CCA TTT AC 3'
**pair2**	
ROP5 CRISPR 3' of locus - for	5' CCA CAA ACT GAA ATC CGA GAG TTT TAG AGC TAG AAA TAG C 3'
Universal sgRNA plasmid primer - rev	see above
**Double CRISPR - Set2**	
**pair1**	
sgRNA cassette KpnI - for	5' CGA ATT GGG TAC CCA AGT AAG CAG AAG CAC GCT G 3'
sgRNA cassette XhoI - rev	5' TCG ACC TCG AGA ATT AAC CCT CAC TAA AGG 3'
**Gibson Cloning - Set3**	
**pair1**	
ROP5 5' homologous region + pUC19 - for	5' TTT CCC AGT CAC GAC GTT CGA CGA AAC AGC AAC TGT GTG 3'
ROP5 5' homologous region + M13F - rev	5' CTG GCC GTC GTT TTA CAA ATT CGG GGG CCG GCT AG 3'
**pair2**	
M13F + ROP5 5' homologous region - for	5' ACT CAC TGA GGA ACC TAG CCG GCC CCC GAA TTC CAA AAA CGA CGG CCA GT 3'
M13R + ROP5 3' homologous region - rev	5' ATG CAC GAA GCA CTA GCA CAG ATA CCG ATG AAC CAG GAA ACA GCT ATG AC 3'
**pair3**	
ROP5 3' homologous region + M13R - for	5' GTC ATA GCT GTT TCC TGG TTC ATC GGT ATC TGT GCT AGT GC 3'
ROP5 3' homologous region + pUC19 - rev	5' AGC GGA TAA CAA TTT CAC ACA TGG TCG AGG CCA TGG TCA 3'
**pair4**	
pUC19 for	5' TGT GAA ATT GTT ATC CGC TC 3'
pUC19 rev	5' AAC GTC GTG ACT GGG AA 3'
**ROP5 KO regions with DHFR*mCherry PCR amp - Set4**	
**pair 1**	
5' ROP5 homologous region - for	5' CGA CGA AAC AGC AAC TGT GTG 3'
3' ROP5 homologous region - rev	5' CAT GGT CGA GGC CAT GGT CA 3'
**ROP5 KO check - Set5**	
**pair1**	
5' of ROP5 5' homologous region - for	5' ACT ATG TCT TTG GTA CGA CCG CCG 3'
DHFR promoter - rev	5' TAC CAG TCA TGG ACG AGA TCG 3'
**pair2**	
DHFR 3' UTR - for	5' ATA ATC TGC GAC CGC TGA ATC CGT 3'
3' of ROP5 3' homologous region - rev	5' GTG ATC CTT GCA GAC GCT TG 3'
**pair 3**	
GRA1 promoter - for (pos control)	5' AAA CCC TCG AAG GCT GCT AGT ACT 3'
GRA1 promoter - rev (pos control)	5' TCT TGC TTG ATT TCT TCA AAG AAC AAC AGC AAG 3'
**ROP18 KO check - Set6**	
**pair1**	
UpUp18 - for	5' GAT CTT CAG CAG CCT GGT TC 3'
DHFR promoter - rev	see above
**pair2**	
DHFR 3' UTR - for #2	5' CGA ACC ATG TCG AGG CTT TAA AGG A 3'
DnDn18 - rev	5' CAA ACC AAT CAC GAC CTC TG 3'
**pair 3**	
GRA1 promoter - for (pos control)	see above
GRA1 promoter - rev (pos control)	see above
